# Proteolytic activity of the nematophagous fungus *Arthrobotrys sinensis* on *Angiostrongylus vasorum* larvae

**DOI:** 10.1186/1756-0500-7-811

**Published:** 2014-11-18

**Authors:** Filippe Elias de Freitas Soares, José Humberto de Queiroz, Fabio Ribeiro Braga, Walter dos Santos Lima, Tatiana Tonini Zamprogno, Jackson Victor de Araújo

**Affiliations:** Departamento de Bioquímica e Biologia Molecular, Universidade Federal de Viçosa, Viçosa, MG Brasil; Departamento de Veterinária, Universidade Federal de Viçosa, Viçosa, MG Brasil; Universidade Vila Velha, Vila Velha, ES Brasil; Departamento de Parasitologia Animal, Universidade Federal de Minas Gerais, Belo-Horizonte, MG Brasil

**Keywords:** Nematophagous fungi, *Angiostrongylus vasorum*, Protease

## Abstract

**Background:**

The predatory nematophagous fungus *Arthrobotrys sinensis* (SF53) produces three proteases with nematicidal activity when grown on solid media culture. However, the proteolytic profile produced by this fungus, when grown in liquid culture medium remains unknown.

**Findings:**

Thus, the objective of this work was to evaluate the production of proteases from nematophagous fungus *Arthrobotrys sinensis* in liquid medium and its nematicidal activity on first stage larvae of *A. vasorum.* Proteases were obtained in its crude form, using Whatman no.1 filter paper, followed by centrifugation for 5 min at 10 × g and 4°C. A zymogram was performed with co-polymerized casein in an acrylamide gel as substrate. An *in vitro* assay to evaluate the nematicidal action of the proteases of *A. sinensis* (SF53) produced in liquid medium on *A. vasorum* L_1_ was conducted. By the analysis of the zymogram, it was observed a single halo at the beginning of digestion of the gel, suggesting that the three proteases of SF53 are produced in an enzymatic complex of large molecular weight. Regarding nematicidal activity, within 24 hours, the proteases produced in liquid medium of *A. sinensis* (SF53) showed a percentage reduction of 64% on the number of L_1_ of *A. vasorum*.

**Conclusion:**

In the present work, it is suggested that the three proteases of SF53 are produced in an enzymatic complex and was also demonstrated that these enzymes were effective in destroying *A. vasorum* L_1_.

## Findings

### Background

*Angiostrongylus vasorum* (Baillet, 1866) Kamensky, 1905, is a protostrongylidae parasite nematode of domestic dogs and wild canids, which causes angiostrongylosis, disease important in public health. In this aspect, stands out the presence of free larvae in the environment and thus the possibility of human infection, since other parasites of the genus *Angiostrongylus* are proven zoonotic. In dogs, the disease is associated with the occurrence of cough, dyspnea, exercise intolerance, weight loss, vomiting, neurological signs, heart failure and death. The infection of dog may occur when ingesting 1) infected paratenic hosts, such as frogs and small mammals, 2) infected intermediate hosts (molluscs) of the genera *Biomphalaria* and *Physa* and among others, 3) or food or water contaminated with free infective larvae in the environment [1, 2].

Despite some successful cases, currently the control of this parasite has been associated with use of anthelmintics that although routinely used, do not act very well on the parasite in the definitive host [3]. Moreover, angiostrongylosis rarely develops acutely and in this sense the clinical signs are perceived later, allowing a continuous environmental dispersion of the parasite through the feces of infected dogs. Some authors have shown that the use of complementary measures to combat helminthoses that complete their development in the environment can be used as tools of control [4, 5]. Thus, the use of nematophagous fungi is cited here. These organisms use mechanical devices such as modified hyphae (traps) and enzymatic artifices to overcome the nematode larvae (production of hydrolytic enzymes, especially proteases) [6].

According to Soares and colleagues [5], one of the promising genera of nematophagous fungi is *Monacrosporium*. Those authors have developed some works that demonstrate their nematicidal activity. In this context, in a recent study, Soares and colleagues [7] showed that the predatory nematophagous fungus *Monacrosporium sinense* (SF53) produces three proteases with nematicidal activity when grown on solid media culture. It is suggested that these extracellular proteases are important at various stages of infection, such as release of nutrients for growth of the microorganism penetration of the cuticle and digestion of the host tissue. However, the production of an enzymatic complex by this fungus remains unclear.

Thus, the objective of this work was to evaluate the production of proteases from nematophagous fungus *Arthrobotrys sinensis* in liquid medium and its nematicidal activity on first stage larvae of *A. vasorum*.

### Materials

The nematophagous fungus *A. sinensis*, isolate SF53, was used for the production of proteases in liquid medium. This isolate is derived from Brazilian soil and has been kept under laboratory conditions through continuous transfer to solid medium. The fungus was cultivated for 10 days in the dark. Then fungal mycelia were transferred to previously autoclaved flasks containing 50 ml of liquid medium composed of (in grams per liter): glucose, 10; yeast extract, 10; K_2_HPO_4_, 5; MgSO_4_, 0.10; ZnSO_4_, 0.005; FeSO_4_, 0.001; CuSO_4_, 0.0005. The inoculum has grown in shaken flasks at 120 × g. After 6 days, proteases were obtained in its crude form, by filtration using Whatman no.1 filter paper, followed by centrifugation for 5 min at 10 × g and 4°C. The supernatant (crude proteases) was used in the subsequent assays [8].

In this study, the strain of *A. vasorum* used has been maintained by the Department of Parasitology, Federal University of Minas Gerais and it is originated from naturally infected dogs, from the city of Caratinga, Minas Gerais [9]. For its obtaining, faeces of infected dogs were collected and placed in a modified Baermann apparatus for the recovery of L_1_. The faeces remained in the apparatus for 12 hours. After this period, the tube was removed, centrifuged at 200 × g for 2 min, the supernatant was discarded and the pellet containing the *A. vasorum* L_1_ was resuspended in 5 ml of 0.85% NaCl. The content present in the tube was homogenized, and from this three aliquots were taken of 10 μL, distributed in glass plate of 7.5 × 2.5 cm. The larvae were counted using a stereomicroscope at increase of 25× [1].

The proteolytic activity was measured by the method of Soares and colleagues [7]. A standard curve of tyrosine was built for the quantification of enzyme activity. One unit of protease was defined as the amount of enzyme required to liberate 1.0 μg of tyrosine per minute under the assay conditions.

A zymogram with co-polymerized casein in an acrylamide gel [10] as substrate (casein-SDS-PAGE) was performed as described by Soares and colleagues [7]. The proteolytic activity was observed by the formation of white halos. The halos of digestion were excised and analyzed by SDS-PAGE (Laemmli, 1970) in order to verify the presence of enzymes.

An *in vitro* assay was conducted to evaluate the nematicidal action of the proteases of *A. sinensis* (SF53) produced in liquid medium on *A. vasorum* L_1_ following the methodology of Soares and colleagues [8]. Two groups were formed in sterile tubes, a treated group containing the crude proteases and a control group (without enzyme), which were then incubated at 26°C in the dark for 24 h. A total of 100 *A. vasorum* L_1_ were poured into sterile tubes containing the crude enzyme. The control group consisted of only 100 *A. vasorum* L_1_ in distilled water. Six replicates were performed for each group. After 24 h, the number of *A. vasorum* L_1_ present in each tube of the treated and control groups was counted.

The data obtained in this experiment were interpreted by analysis of variance in significance levels of 1 and 5% probability. The efficiency of L_1_ predation compared to control was assessed by the Tukey test at 1% probability [11]. Subsequently, the percentage reduction of average larvae (L_1_) was calculated according to the following equation:


### Results and discussion

**Figure 1:**
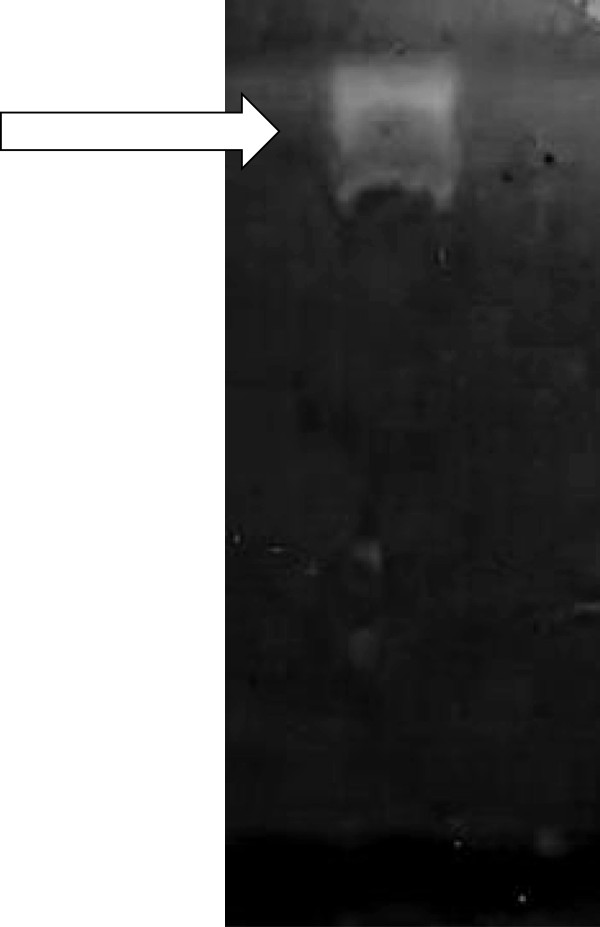
**Zymogram of the proteases of** ***Arthrobotrys sinensis*** **(SF53) in liquid medium.** Zymogram of the proteases produced by *Arthrobotrys sinensis* (SF53) in liquid medium. Through analysis of the zymograms, a single halo of digestion at the beginning of the gel was observed, suggesting that the three proteases of SF53 are produced in an enzymatic complex of large molecular weight.

It was observed that the fungus *A. sinensis* (SF53) has produced proteases, when grown in an inducer liquid medium. However, proteolytic activity (15.78 U/mL) was lower than that obtained when the same fungus was grown on solid culture medium (38.0 U/mL) [7]. Moreover, a single halo was observed at the beginning of digestion of the gel, suggesting that the three proteases of SF53 are produced in an enzymatic complex of large molecular weight (Figure 1). When grown on solid culture medium, three evident halos were observed in the zymogram of the same fungus [7]. Probably this difference is due to the enzymatic extraction by stirring, a more brute technique, in the case of solid medium, which may have ruptured the enzymatic complex, “releasing” each protease.

Regarding nematicidal activity, within 24 hours, the proteases produced in liquid medium of *A. sinensis* (SF53) showed a percentage reduction of 64.3% on the L_1_ of *A. vasorum*. Also, difference (p <0.01) was observed in nematicidal action in relation to larvae present in the control group, in the same studied interval.

The predatory activity of fungi of *Monacrosporium* genus has been tested on *A. vasorum* L_1_. However, only one early work [12] had demonstrated its capture and subsequent *in vitro* destruction, in culture medium WA2%. Braga and colleagues [12] demonstrated that isolate SF53 was effective (p < 0.05) in the capture and destruction of *A. vasorum* L_1_ under laboratory conditions, registering at the end of seven days the percentage reduction of 74.2%. However, in the present work, it was noted that the obtained percentage reduction was 64.3%, what is interesting from a biological point of view, since only enzyme was used (no fungi).

**Figure 2:**
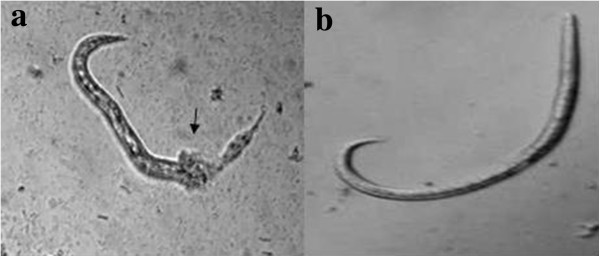
**Nematicidal activity of** ***Arthrobotrys sinensis*** **(SF53) proteases. (a-b)** Photomicroscopy of nematicidal activity of proteases from nematophagous fungus *Arthrobotrys sinensis* (SF53) on first stage larvae of *Angiostrongylus vasorum* after 24 hours (treated group **(a)** and control group **(b)**).

Furthermore, our results suggest that the nematicidal activity of SF53 was due to the action of enzymes on the cuticle of the L_1_ of *A. vasorum*, since the cuticles of larvae are especially rich in proteic components that hinder the action of antagonist organisms (Figure 2). Accordingly, another work developed by the present group showed that the use of crude enzyme extract of nematophagous fungus on *Ancylostoma caninum* L_3_ (a geohelminth) showed good efficacy [13]. In that work, it was observed the hydrolysis of the cuticle by enzymatic action, which also has acted inside the nematode, causing its destruction.

Soares and colleagues [7] reported that extracellular proteases are an important virulence factor for the species *M. sinense*. Its nematicidal activity was also evaluated on *Panagrellus redivivus* larvae (free-living nematode), and at the end of the experiment the average percentage was 79% of reduction in the number of recovered larvae. In the present work, another enzyme production was tested using liquid culture medium, and the results were interesting both for proteolytic and nematicidal activity. Furthermore, it is also suggested that the use of *A. vasorum* can probably contribute to further research about its control.

Three proteases were produced in both cases (solid and liquid medium), however, probably because of differences in the extraction process (much more gentle in the case of liquid medium, using only filtration and centrifugation, than in the case of the solid medium in which there is an intense mechanical agitation), we can find at beginning of the gel a halo that suggests the presence of an enzymatic complex of large molecular mass, which has failed to migrate into the gel, due to this large mass.

In the present work, three proteases of the isolate SF53 were produced in an enzymatic complex and was also demonstrated that these enzymes were effective in destroying *A. vasorum* L_1_ under laboratory conditions.

## Authors’ information

Jackson Victor de Araújo CNPq scholarship.
